# Antimicrobial use of patients with sexually transmitted infection symptoms prior to presentation at five health facilities in Southern Ghana

**DOI:** 10.1186/s13756-023-01351-8

**Published:** 2023-12-13

**Authors:** Naiki Attram, Helena Dela, Eric Behene, Nicholas N.A. Kyei, Karen Ocansey, Jennifer N. Yanney, Edward O. Nyarko, Kennedy K. Addo, Kwadwo A. Koram, Anne Fox, Andrew Letizia, Terrel Sanders

**Affiliations:** 1 Naval Medical Research Unit-EURAFCENT, Accra, Ghana; 2 Noguchi Memorial Institute for Medical Research, University of Ghana, Legon, Ghana; 3 Ghana Armed Forces Health Directorate, 37 Military Hospital, Accra, Ghana; 4 Institute of Public Health, Charité – Universitätsmedizin Berlin, Berlin, Germany; 5 Heidelberg Institute of Global Health, Heidelberg University, Im Neuenheimer Feld 324, 69120 Heidelberg, Germany

**Keywords:** Antimicrobial use, Antimicrobial resistance, Antimicrobial stewardship, Gonorrhea treatment

## Abstract

**Background:**

Unregulated and inappropriate antimicrobial use are major contributors to the evolution of antimicrobial resistance worldwide. It is important to monitor and collect data on the use of antibiotics at health facilities and in the general population in order to support antimicrobial stewardship programs.

**Methods:**

As part of a gonorrhea surveillance study that was conducted from June 2012 to Jan 2018, we administered a questionnaire to elicit information on the types of antimicrobials used by individuals to treat symptoms of a gonorrhea infection prior to presenting at five health facilities in Southern Ghana.

**Results:**

Almost one-third (383/1,349; 28%) of study participants admitted taking one or more antimicrobial types before hospital presentation, while 138/383 (36%) of those who took antimicrobials could not remember what they ingested. A greater percentage of individuals who reported prior antimicrobial use before presentation at a health facility tested positive for gonorrhea by NAAT (30%), in contrast to 24% for those without prior treatment (p = 0.004). Penicillin and its derivatives, as well as ciprofloxacin and doxycycline, were the most used, while a few individuals reported taking drugs such as kanamycin and rifampin. Males were more likely than females to take an antimicrobial prior to attending a health center.

**Conclusion:**

In order to curb excessive and inappropriate antimicrobial use, antibiotics used by patients before presenting at hospitals ought to be investigated by healthcare providers. It is recommended that health professionals receive continuing education on the consequences of unregulated antimicrobial use.

**Supplementary Information:**

The online version contains supplementary material available at 10.1186/s13756-023-01351-8.

## Background

The World Health Organization (WHO) considers the rising trend of AMR as one of the top threats to global public health systems [1]. The direct impact of AMR on healthcare is severe [2, 3]. Global annual mortality from AMR has been estimated at 700,000 [4]. Consistent monitoring of AMR rates in low- and middle-income settings is scarcely done [5]. However, the available data indicate that AMR rates in several disease pathogens have been increasing worldwide [6–9]. Unregulated use of medicines strongly contributes to the release of antimicrobials into the environment [1, 4, 8].

Currently,there is no known effective drug on the market for empirical gonorrhea monotherapy [10]. Ceftriaxone-azithromycin, one of the combination drugs recommended by the WHO for empirical treatment of gonorrhea, was recently reported to have failed to treat gonorrhea infections in England and Australia [11–13]. Additionally, treatment failure to a ceftriaxone-doxycycline combination was reported in France in 2017 [14]. Suspected cases of gonorrhea in Ghana are usually treated with cefixime or ceftriaxone with azithromycin or doxycycline, although ciprofloxacin is recommended on a case-by-case basis as per the Ghana Standard Treatment Guidelines [15], [clinician, personal communication]. Data on antimicrobial use can be applied to support stewardship programmes that help to counter the spread of AMR. This study sought to comprehensively investigate and analyze the patterns of prior antimicrobial use among patients seeking treatment for urethritis or cervicitis symptoms, focusing on the prevalence, types, and sources of antimicrobials, as well as the association between prior treatment and the incidence of gonorrhea, to inform targeted interventions for improved antimicrobial stewardship and effective management of sexually transmitted infections.

## Methods

Inclusion criteria comprised one or more of the following symptoms: urethral or vaginal discharge, painful urination, pain or swelling in one testicle (males), painful intercourse, and post-coital bleeding (females). Patients who visited five health facilities from June 2012 to January 2018 in the Southern part of Ghana with symptoms of urethritis as described above were enrolled in a study approved by the following Institutional Review Boards: Noguchi Memorial Institute for Medical Research, 37th-Military Hospital, Ghana Health Service, and the Naval Medical Research Centre. Four of the health facilities were part of a gonorrhea surveillance study and were selected based on their involvement with the study authors in prior surveillance studies [16, 17]. The fifth health facility is a Sexually Transmitted Diseases care center with attendance of high-risk groups like men who have sex with men and commercial sex workers. Written informed consent was obtained from each participant as previously described [16]. A nucleic acid amplification test (NAAT) was performed to detect *N. gonorrhoeae* in patients’ urine specimens, and bacterial culture was attempted on each genital swab specimen [17]. Additionally, a questionnaire was used to collect data on demographics and antimicrobial use, among other information. Condom use, alcohol use, and other risk factor data were collected and reported elsewhere [17].

The Chi-square test was used to determine associations between sex with age group, education, and self-reported treatment of the study participants. The Chi-square or Fisher’s exact tests were used to determine associations between sex and the type of antimicrobials taken.

## Results

The study involved the participation of 1,349 patients, with a breakdown of 1,339 participants of known gender: 60% (805) identified as female, while 40% (534) identified as male. Most study participants were aged 18–38 years (89%, n = 1194) and had some formal education (96%,n = 1276). Notably, 28% (383) acknowledged using one or more antimicrobials/herbal drugs before seeking medical attention at the health facility.

No statistically significant association was observed between educational level and self-reported prior use of antimicrobials. However, there was a strong statistical association seen between sex and self-reported prior treatment with antimicrobials (*p* < 0.001) (Table 1).

**Table 1:** Demographic and prior-drug use information of study participants

	Total N(%)	Male^a^ n(%)	Female^a^ n(%)	*p-value*
**Age group** ^**b**^				0.233
18–24 years	384(29.0)	138(26.2)	246(30.6)	
25–31 years	553(41.7)	234(44.0)	319(39.7)	
32–38 years	257(19.3)	98(18.6)	159(19.8)	
39–45 years	96(7.2)	37(7.0)	59(7.3)	
46 years and above	41(3.1)	20(3.8)	21(2.6)	
**Educational level** ^**c**^				0.283
No education	50(3.8)	18(3.4)	32(4.0)	
Primary/Junior High School	238(18.0)	85(16.1)	153(19.2)	
Secondary and above	1038(78.3)	425(80.5)	613(76.8)	
**Antimicrobial use within 2 weeks** **prior to clinic presentation**				< 0.001
Yes	383(28.4)	201(37.6)	182(22.6)	
No	966(71.6)	333(62.4)	623(77.4)	

^a^Out of a total of 1349 enrolees, 10 patients are missing data on sex

^b^18 patients missing data on age

^c^23 patients missing data on the educational level

*p*-value was obtained via the chi-square test

**Fig. 1:**
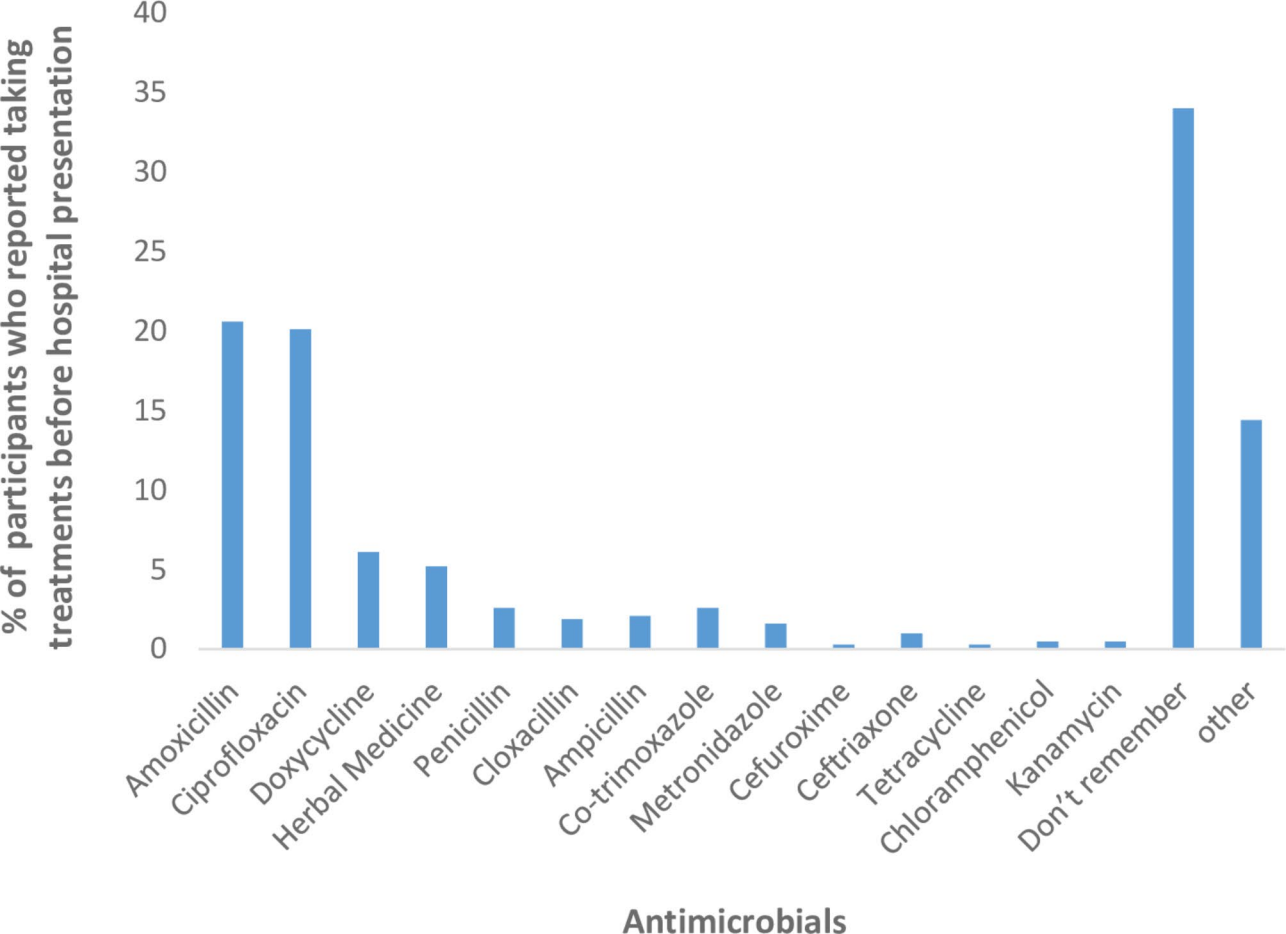
**Description of the most commonly used antimicrobials by study participants before hospital presentation (N=383)** **This figure includes multiple responses**

Approximately two-thirds (66%,n = 252) of the people who took antimicrobials/herbal drugs before presenting at the health center remembered the names of the drugs they ingested. The most consumed antibiotics were amoxicillin, ciprofloxacin, and doxycycline, representing almost half (47%,n = 179) of the drugs taken. Penicillin-based drugs represented nearly a third (27%,n = 104) of the drugs used (Fig. 1).

A minority of those seeking treatment before reaching the health center opted for herbal medicine (5%). In contrast, a minimal percentage, specifically 0.2% (1) and 1% (4), underwent treatment with cefuroxime and ceftriaxone, respectively—both drugs recommended for empirical gonorrhea treatment. On the other hand, the remaining study participants reported the use of drugs not approved for gonorrhea treatment. These drugs included kanamycin, metronidazole, trimethoprim-sulfamethoxazole, chloramphenicol, potassium citrate, co-trimoxazole, secnidazole, rifampin, and cetirizine.

There were no statistically significant associations observed between the sex of the study participants and the antimicrobial ingested, except for those who took amoxicillin. However, a noteworthy association was identified between the sex of the study participants and their ability to recall the specific drug they had ingested (Table 2).

**Table 2:** **Antibiotics and other medication taken by patients within two weeks prior to hospital presentation**

Type of antibiotic or treatment	Total^a^ (N%)	Male (n%)	Female (n%)	*p-value*
Amoxycillin	79(20.6)	32(15.9)	47(25.8)	0.022^b^
Ciprofloxacin	77(20.1)	43(21.4)	34(18.7)	0.526^b^
Doxycycline	23(6.1)	10(5.0)	13(7.1)	0.397^b^
Herbal medicine	20(5.2)	13(6.5)	7(3.9)	0.358^c^
Penicillin	10(2.6)	7(3.5)	3(1.7)	0.343^c^
Trimethoprim/sulfamethoxazole	10(2.6)	5(2.5)	5(2.8)	1.000^c^
Ampicillin	8(2.1)	5(2.5)	3(1.7)	0.726^c^
Flucloxacillin	7(1.8)	4(2.0)	3(1.7)	1.000^c^
Metronidazole	6(1.6)	1(0.5)	5(2.8)	0.106^c^
Ceftriaxone	4(1.0)	1(0.5)	3(1.7)	0.350^c^
Chloramphenicol	2(0.5)	2(1.0)	0(0.0)	0.500^c^
Kanamycin	2(0.5)	2(1.0)	0(0.0)	0.500^c^
Cefuroxime	1(0.3)	0(0.0)	1(0.6)	0.475^c^
Tetracycline	1(0.3)	0(0.0)	1(0.6)	0.475^c^
Others	55(14.4)	28(13.9)	27(14.8)	0.884^b^
Cannot remember drug name	138 (36)	82(40.8)	56(30.8)	0.041^b^

^a^Total drugs taken do not add up to the number of patients because some reported taking more than one antimicrobial

^b^p-value was obtained via the chi-square test

^c^p-value was obtained via Fisher’s exact test

A majority of study participants (72%) reportedly did not take any drugs, as compared to 28% who admitted to receiving medication before hospital presentation. Notably, a higher percentage of those who underwent treatment before presentation tested positive for gonorrhea by NAAT (30%), contrasting with 24% for those who did not receive prior treatment (p = 0.004).

**Fig. 2:**
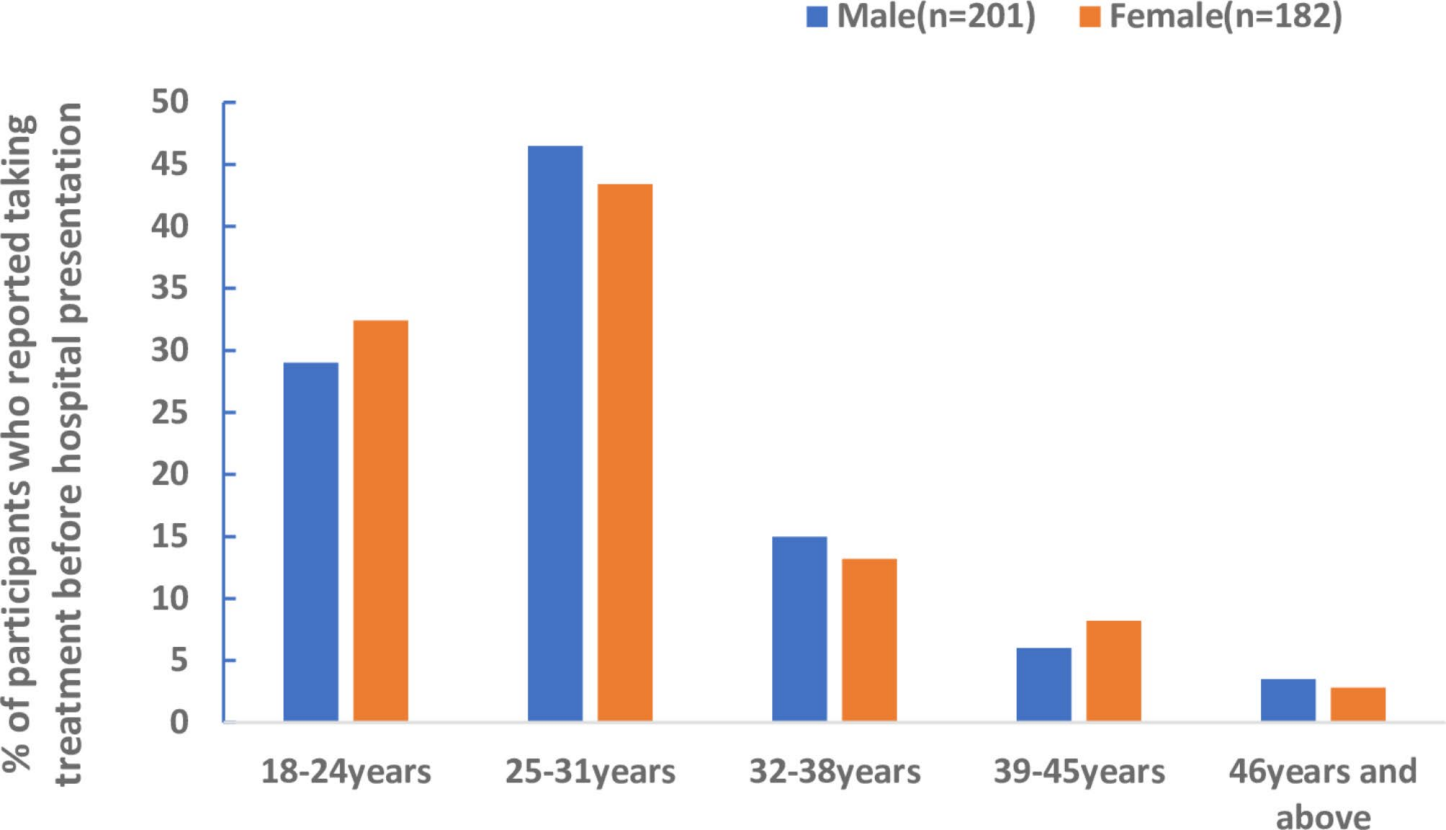
**Trends of antimicrobial use within age groups stratified by sex**

There was no significant difference between the age groups of the study participants and their self-reported antimicrobial intake before hospital presentation (*p* = 0.802) (Fig. 2).

Among the study participants, some took antimicrobials in combination as follows: ciprofloxacin with doxycycline (4%), ciprofloxacin with metronidazole (2%), ciprofloxacin with co-trimoxazole (1%), ciprofloxacin with amoxicillin (0.4%) and ciprofloxacin with penicillin (0.4%). Almost a fifth (18%) of those who reported antibiotic treatment took two or more types in combination.

Among the study participants who reported taking medication before hospital presentation, 50% obtained the drugs from a pharmacy, 36% obtained their drugs from a doctor, and 7% each from a traditional healer and friends .

## Discussion

This study investigates prior antimicrobial use among patients seeking treatment for urethritis symptoms to characterize and inform targeted interventions for improved antimicrobial stewardship and effective management of sexually transmitted infections. The key findings of this study offer valuable insights into the dynamics of prior antimicrobial use and its implications. Approximately one-third of individuals reported engaging in prior antimicrobial use before presenting at a health facility, with a noteworthy preference for specific antimicrobials such as amoxicillin, ciprofloxacin, and doxycycline. Less than a quarter of patients consumed two or more types of antimicrobials during prior-treatment, while the rest consumed just one type. Pharmacies emerged as the predominant source for obtaining these medications. We also observed an interesting association between prior antimicrobial use and a higher incidence of gonorrhea positivity, particularly evident in individuals who engaged in prior-treatment.

The revelation that approximately one-third of patients sought treatment for urethritis or cervicitis symptoms after engaging in prior antimicrobial use underscores a significant aspect of healthcare-seeking behavior in this population. This finding suggests a noteworthy level of autonomy in healthcare decisions in this population, highlighting the need for targeted patient education initiatives. A prevailing belief in Ghana indicates that self-medicating with purchased drugs is quicker and more cost-effective, particularly for minor ailments [18]. Thus, a significant proportion of individuals first treat their genital infections and only resort to clinical care when there is no improvement in their health condition. Data from this study also suggests that this tendency is demonstrated more in males than in females. Emphasizing the importance of seeking professional medical advice and testing before procuring medications.

The types of antimicrobials chosen by the patients also merit closer scrutiny. The identification of specific antimicrobials, such as amoxicillin, ciprofloxacin, and doxycycline, raises questions about the appropriateness of these choices for the targeted symptoms. These antibiotics were historically employed for gonorrhea monotherapy but have gradually lost effectiveness [19]. Current guidelines recommend dual therapy involving cefixime or ceftriaxone in conjunction with azithromycin or doxycycline, as endorsed by the World Health Organization [20].

Despite being classified in 2017 as prescription-only medicines by Ghana’s Food and Drugs Authority (FDA) [21], half of the drugs reportedly used in this study were obtained from pharmacists, raising concerns about adherence to existing regulations. In Ghana, the Health Professions Regulatory Body Act (857) of 2013, stipulates that only specific healthcare professionals should prescribe antimicrobials. However, legal structures to enforce adherence do not exist. Unauthorized groups, including licensed chemical sellers and traditional practitioners, contribute to the unauthorized sale of antibiotics [22].

Identification of pharmacists as the primary source for obtaining antimicrobials for symptomatic treatment of urethritis or cervicitis aligns with previous research and signifies a persistent and crucial aspect of healthcare access and medication distribution [23]. The convenience and accessibility of community pharmacies, especially for individuals seeking treatment for potential sexually transmitted infections, may contribute to the high percentage observed in this study. The influence of pharmacies on patient decision-making is noteworthy, and understanding the factors that drive patients to choose pharmacies is essential.

The unregulated use of antimicrobials in LMICs, such as Ghana, is influenced by factors like a lack of awareness regarding the long-term consequences of unchecked antimicrobial use on AMR, a shortage of qualified pharmaceutical staff, and economic pressures on drug retailers [24]. Administering antimicrobials before diagnostic evaluations may impede pathogen growth, leading to false-negative culture results and complicating treatment decisions [25]. Additionally, this practice may prove ineffective against the pathogen due to antimicrobial resistance, resulting in the unwarranted and excessive use of antibiotics. Notably, our study revealed significantly higher gonorrhea Nucleic Acid Amplification Test (NAAT) positivity among individuals reporting antimicrobial use before presenting at a health facility compared to those without prior treatment. This substantiates the ineffectiveness of most antimicrobials consumed before hospital presentation in treating the underlying infection. The AMR surveillance data gathered from the study participants detailed in this research reveals a concerning scenario with prevalence rates exceeding 80% for resistance to tetracycline, penicillin, and ciprofloxacin among *N. gonorrhoeae* specimens collected throughout the study period [17]. This underscores the critical necessity for heightened awareness among the Ghanaian public regarding the judicious use of antibiotics. The case of gonorrhea infections serves as a poignant example, signaling that the pathogen is teetering on the verge of becoming untreatable due to escalating antimicrobial resistance against commonly employed antibiotics. Urgent and concerted efforts are imperative to promote responsible antibiotic usage and curb the dangerous rise of resistance in the context of STIs.

Several limitations should be considered in interpreting the findings of this study. Firstly, the study questionnaire did not comprehensively address whether participants obtained antimicrobials with a prescription, leaving a gap in understanding the regulatory context of antimicrobial access. Additionally, the absence of information on whether study participants had been referred from other healthcare centers limits the contextualization of antimicrobial self-use among patients with urethritis/cervicitis. Furthermore, the study’s data was derived from a specific subset of the Ghanaian population, potentially impacting the generalizability of the findings to a broader demographic. Nonetheless, while confined to individuals exhibiting symptoms of Sexually Transmitted Infections (STIs) within a specific segment of the Ghanaian population, the findings from this study align with prior research on antimicrobial utilization across diverse patient cohorts. For instance, a study encompassing patients awaiting laboratory tests in the Ashanti and Eastern Regions, primarily for conditions like diarrhea, aches, pains, or urinary tract infections, reported a similar trend, with approximately a fifth disclosing prior antimicrobial use before seeking medical attention [8]. Notably, in that study, urine bioassays revealed a significantly higher antimicrobial activity rate (31.6%) than the self-reported treatment rates but comparable to the self-reported treatment rates observed in the current investigation. Unfortunately, the present study did not perform antimicrobial activity tests of patients’ urine for comparison. Such assay results could have helped confirm the reported antimicrobial use of study participants. These limitations underscore the need for caution in extrapolating the results and emphasize avenues for more extensive investigation, including a nuanced exploration of prescription practices and referrals in future studies.

## Conclusion

The utilization of penicillin-based drugs, ciprofloxacin, and trimethoprim-sulfamethoxazole for empirical gonorrhea treatment in Ghanaian health facilities should be reassessed. Investigating prior antimicrobial intake, promoting reliance on laboratory test results for treatment decisions, and conducting targeted public education, especially for males with urethral discharge, can curb unsupervised antimicrobial use. Continuous education for healthcare providers, informed by available AMR surveillance data, will ensure the judicious use of recommended antimicrobials in empirical treatment. Finally, strengthening law enforcement capabilities is vital for addressing non-compliance with established regulations and policies governing antimicrobial importation, sale, and usage. This multifaceted approach will enhance treatment efficacy, reduce AMR, and promote responsible antimicrobial practices.

### Electronic supplementary material

Below is the link to the electronic supplementary material.

Supplementary Material 1

## Data Availability

The data supporting the findings of this study are available within the paper and its Supplementary Information. The raw data however, are available from the corresponding author upon reasonable request and are located in controlled access storage.
