# Supplementary material for: Antimicrobial use of patients with sexually transmitted infection symptoms prior to presentation at five health facilities in Southern Ghana
Source: Antimicrob Resist Infect Control. 2023 Dec 13;12:146. doi: 10.1186/s13756-023-01351-8 (PMC10720218; doi:10.1186/s13756-023-01351-8)
Supplement: Supplementary file 1 — Supplementary Material 1 [file 13756_2023_1351_MOESM1_ESM.docx]

**Supplementary File 1.**

**Sexually Transmitted Diseases Surveillance in five clinics in Accra and Sekondi/Takoradi** “Patient Questionnaire” Date:…../…../……

| We want to ask you a few questions about sexually transmitted diseases, your participation is voluntary and you may stop at any time while answering the questions. Many questions will assess your knowledge, behaviors, and the environment you live in that may have contributed to cause the symptoms you have. This kind of information will be useful in planning of public health programs in Ghana and countries within the sub region.  **Demographics** | |  |  |  |
| --- | --- | --- | --- | --- |
| 1. Age in years __ __ | 2. Gender:  Male  Female |  |  |  |
| 3. Marital Status:  Single  Married  Divorced  Widowed  Separated | |  |  |  |
| If married before, how old were you when you first got married? _______________________ | |  |  |  |
| 4. Education:  No school  Primary  JHS  SHS and above | |  |  |  |
| 5. Occupation: Military, Rank _______________________ Civilian _________________________________ | |  |  |  |
| 6. Ethnicity: ___________________ | |  |  |  |
| 7. Religion: : Christian Muslim Traditionalist No religion Other___________________  8. Where do you live ______________________________________________ | |  |  |  |
| **Present History, Behavior, Risk factors** | | |  |  |
| * **Skip (Q9&10) if respondent is a male**:  9. Are you pregnant  Yes  No  10. Are you on birth control pills  Yes  No  11. How often do you travel?  Weekly  Monthly  Occasionally Other _________________________ | | | | |
| 12. What do you travel for?  Work  Funeral Visit/vacation  Other________________ | | | | |
| 13. Do you have sexual encounters when you travel?  Yes  No | | | | |
| 1. What symptoms do you have today**? Check all that apply** :   Burning during urination  Discharge from penis (male) or vagina (female )  Bleeding from penis (male) or vagina (female)  Foul smell/odor from urine Pain in penis (male) or abdomen (female)  Pain when having sex  Ulcers on genital parts  Genital warts  Other describe: ______________  15. How many days have you had the symptoms _______________  16. Have you taken any medication in the past 2-weeks?  Yes  No IF Q16 IS NO, SKIP TO 21  17. Which medication did you take?  Antibiotics _______________________________  Painkillers ____________________________  Other ______________________________________  18. Did you complete your dosage?  Yes  No IF ANSWER IS YES SKIP TO Q20  19. What was your reason for non- compliance?  I got better  Antibiotics did not work  I was experiencing adverse side effects  Other__________________________  20. Who gave you the medication?  Doctor  Pharmacist  Friend/Family  Traditional healer  Other Describe: ……………………… | | | | |
| 21. Do you drink alcohol/ beer?  Yes  No **(If no go to Q 24)**  22. How often do you?  Daily  Once a week  Twice a week  Occasional  Other____________________ | | | | |
| 23. How much do you drink a day?  Bottle  Tot Quantity____________________  **(Indicate if Bottle/tot and the quantity of bottle/tot)** | | | | |
| 24. How many sexual partners have you had in the last 6 months?  One  Two  Three and above  25. Have you had a new sexual partner in the past month  Yes  No  26. Do you currently have more than one sexual partner  Yes  No  27. Does your partner have other sexual partners  Yes  No  Don’t know  28. At what age did you become sexually active (first sexual encounter) ________________________________ | | | | |
| 29. How often do you use a condom?  Never  Always  when I have a new partner  At the discretion of my partner  When I question the fidelity of my partner  Other____________________ | | | | |
| 30. Have you been diagnosed with STI in your lifetime?  Yes  No  31. If Yes, what type of STI were you diagnosed with?  Syphilis  Chlamydia  Gonorrhea  HIV  HPV(warts)  Other_______________________________________  **(If Q31 response is HIV then CONTINUE TO Q37-Q39, OTHERWISE SKIP Q37-39 AND GO TO Q40)**  32. How many times have you had this condition?  Once  Twice  Thrice  Other___________  33. Have you had this condition in past six months?  Yes  No  34. Were you diagnosed in the lab before treatment?  Yes  No  35. How were you treated? _________________________________________________________________  36. Did you complete your treatment?  Yes  No **GO TO Q39-Q43** | | | | |
| 37. When did you go for the HIV test?  Less than 3months ago  Between 3-6months ago More than 6 months ago  38. Are you receiving treatment?  Yes  No  39. Are you taking the medication as prescribed?  Yes  No | | | | |
| 40. Do you have any chronic health condition?  Yes  No  41. If yes which condition? _____________________________  42. Are you on medication for this illness?  Yes  No  43. Which medication? _____________________________  44. How often do you take medication?  Daily  Once a week  Twice a week  Occasionally  Other_____________________________  **(END INTERVIEW)** | | | | |

Comments:

___________________________________________________________________________________________________________________________________________________________________________________________________________________________
